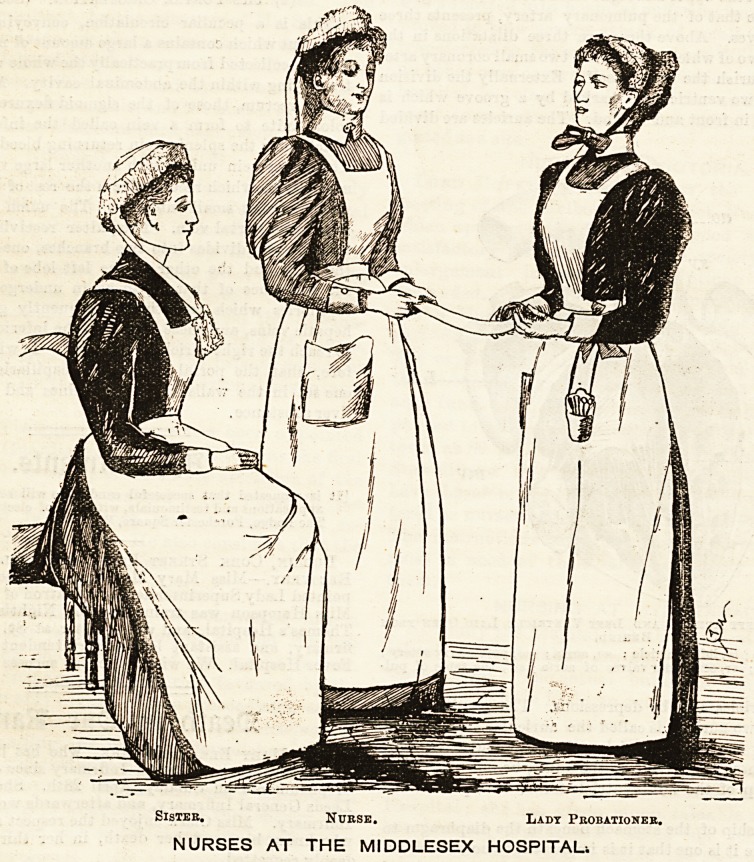# The Hospital Nursing Supplement

**Published:** 1895-05-11

**Authors:** 


"The Hospital" Nursing Mirror.

Being the Extra Nursing Supplement of "The Hospital" Newspaper.

[Contributions for this Supplement should be addressed to the Editor, The Hospital, 428, Strand, London, W.C., and should have the word "Nursing" plainly written in left-hand top corner of the envelope.]

### News from the Nursing World.

### OUR PRINCESS.

Many readers will rejoice at the prospect of seeing Her Royal Highness the Princess of Wales on June 27th, when she has graciously consented to open the grand bazaar at the Portman Rooms. The proceeds of the bazaar will be devoted to St. Mary's Hospital, Paddington, of which His Royal Highness the Duke of York is president. The Clarence Memorial Wing at the same institution is a material proof of the interest exhibited by many members of the Royal Family in this popular hospital.

### FIRE AT A FEVER HOSPITAL.

The fire which broke out in the North-Western Fever Hospital at Hampstead on the 6th inst. was, happily, confined to the laundry and drying block. Considerable damage was caused to the structure, but beyond a temporary alarm it appears that the patients were not inconvenienced. The fire took place in the afternoon and its cause has not transpired.

### WARDS AND WASHING.

Circular wards are not always popular with patients, whilst charge nurses find difficulties in supervising the occupants of the beds, two or three of which are at times just outside their range of vision. Especially at night does the conscientious nurse feel the added anxiety of working in a ward where the central pillar obstructs her view of her charges. But objections to circular wards vanish from the mind of the visitor to Hampstead Workhouse Infirmary when tea-time arrives. This meal is taken by patients "allowed up" at tables which come out from the centre like the spokes of a wheel. Parties of four or six sit round in a home-like fashion, most unsuggestive of pauperism. The snowy linen is another pleasant feature at this infirmary, although the washing for it and the workhouse itself is done in a laundry of modest dimensions. The caps of the poor old "grannies" and the sheets and shirts of the men have a most creditable appearance. The drying ground borders a terrace garden gay just now with spring flowers, where the old people sit and bask in the sun. A large perambulator discharging a load of bonnie children in the entrance hall showed that rides over the Heath form part of the treatment of juvenile inmates, whose appearance certainly suggested healthful environments.

### ST. LUKE'S HOUSE.

In Osnaburgh Street, near to Portland Road Station, stands a house within the walls of which good work is unostentatiously accomplished. Before entering the door a visitor notices the window-boxes filled with gay blossoms, the clean panes, fresh curtains, and spotless brass handles, and the plate bearing the name of "St. Luke's House." It cannot be easy to maintain this bright cleanliness in such a locality, yet the little house looks as dainty as when it was first opened. The patients admitted are very hopeless ones, often discharged from general hospitals, unsuitable for further treatment, or such as are inadmissible to most other institutions. St. Luke's House was established specially for such cases, and the little wards are peopled respectively with men and women in the latest stages of incurable disease. Occasionally a patient's general health improves so much with medical treatment, sanitary surroundings, and good food that he elects to go home again, temporarily relieved and disproportionately sanguine. The majority, however, remain under kindly care at St. Luke's House, until death ends the sufferings which skill and tenderness have done much to lessen even in cases of cancer and phthisis. By the removal of partitions, and other ingenious devices, the rooms have been adapted to their present use, and although a spare inch of space does not exist, the best has been made of somewhat limited accommodation. The lady superintendent, Miss Petrie, may well be congratulated on the comfort which she has been instrumental in giving to many. Particulars as to admission can be obtained from her.

### A COTTAGE HOSPITAL.

In a short half-hour the train bears the traveller from the noise and bustle of King's Cross to the peaceful country surroundings of the Cottage Hospital at Potters Bar. That pretty institution, flooded with light from numerous windows, benefits by the pure air of the locality. The cleanliness which pervades the whole place, and the home-like comfort enjoyed by the inmates are specially noteworthy, and the matron shows herself a good housekeeper as well as a skilful nurse by her attention to every detail of management. Many minor casualties are brought to the hospital as well as graver accidents and illnesses. The dispensary or surgery is a room well adapted for emergency cases. The laundry is a detached building lying a short distance from the house, and the day-room for the convalescents has a pleasant outlook and well-furnished book-shelves. The hospital accommodates ten patients, and when there are bad cases the matron and her probationer divide the night-work between them. This arrangement is never desirable, and urgent cases requiring night-nursing should have it supplied by an extra nurse, who could be temporarily engaged.

### THE GUARDIANS OF THE POOR.

Such undignified squabbles took place at a recent meeting of the Yarmouth Board that it appears as if the interests of the sick had been quite overlooked by those who are the ostensible guardians of the poor. The suggested inquiry by the Local Government Board appears not only desirable but necessary, for the disputes reached a point at which even the engagement of a successor to the nurse who has left was actually postponed.

### QUEEN'S NURSE AT WELLINGBOROUGH.

The Queen's nurse secured to the sick poor of Wellingborough by the energy of the St. John Ambulance Brigade is doing excellent work. The committee are well pleased to find how thoroughly acceptable Nurse Pickering's services have proved. She has attended 148 cases in the course of the year, many of them very serious ones.

### DISTRICT NURSES IN SOMERSET.

The Taunton District Nursing Association has issued its annual report, and the services of Nurses Pike and Sage appear to have been greatly appreciated by their patients. The Maternity Branch has proved particularly valuable, and Nurse Watling has attended 207 cases, paying them 2,835 visits. The Samaritan Fund has proved a valuable auxiliary to the association, and various gifts of nursing appliances are gratefully acknowledged by the committee. The nurses are permitted, by the courtesy of Mr. Saunders and Mr. Perkins, to use their omnibuses without payment, a privilege of which they must be often glad to avail themselves. The rules and bye-laws of the association seem very reasonable, and the nurses are neither allowed to become almoners nor charwomen.

### NURSES' EARNINGS.

The financial statement laid before the Quarterly Court of Governors of the Devon and County Hospital shows a considerable balance in hand on the earnings of private nurses sent out by the institution. The accounts published in the local press do not state whether any of this money is to profit the nurses. Perhaps the annual report may explain the destination of the sum of over £240, which should, of course, be invested towards pensions for the private nurses, or be used in some other way for their personal advantage.

### LEWES.

A Ladies' Visiting Committee has been appointed by the Lewes Guardians, with Miss Bacon as the first chairman. Dr. Crosby has called the attention of the Board to the need of a trained night nurse in the infirmary, and reports that more assistance is required for helpless patients. He also considers that the diets require some modifications.

### A FETE AT DERBY.

The drill hall at Derby was converted into a quaintly realistic representation of an old English market place on the occasion of the recent bazaar. The sixteenth century was the period favoured by the committee who ably organised the proceedings, and a charming result was effected. The picturesque old houses still standing, or recently demolished in the town, were reproduced with fidelity, and the furnishing fund of the Derbyshire Royal Infirmary cannot fail to have been largely augmented by the energy and taste which created this novel fête for its benefit.

### STOCKTON AND THORNABY.

The acquisition of a permanent convalescent home by the Stockton and Thornaby Nursing Association is a plan likely to receive prompt and substantial encouragement in the district. The workmen, realising the benefits derived from the home rented for the use of convalescents last season, are anxious to co-operate as far as they can in the present scheme. The Nursing Association appears well managed and prosperous, and a fifth district nurse is about to be added to the staff. The resignation of the lady superintendent, Miss Barker, was recorded at the annual meeting with universal regret. The exertions of the President, Lady Londonderry, in promoting the trained nursing of the poor in their own homes have certainly been crowned with success.

### QUARANTINE AT MELBOURNE.

A veritable small-pox scare occurred in Melbourne this spring following the arrival of a trading vessel with a Lascar crew from China. There were several cases of small-pox on board, and the ship was quarantined, officers vaccinated, luggage and effects fumigated under the superintendence of the health officers. No new cases occurring, the people were released after the usual time had elapsed. The second officer took lodgings in Melbourne, and twelve days later his landlady was admitted into hospital suffering from a fever which proved to be a severe attack of small-pox. She was transferred to the sanatorium near Williamstown, and no other case occurred. The erection of an infectious hospital is being urged upon the authorities, and a coast town near the South Australian border is suggested as a site.

### NURSING IN VICTORIA.

Lord Hopetoun presided at the tenth annual meeting of the Melbourne District Nursing Society, which appears to be doing much good work. For the satisfactory maintenance of the society and for its enlargement increased subscriptions are urgently demanded. The first annual report of the St. Vincent's Hospital shows the number of cases treated as 2,584 during the year, 441 being indoor patients. The expenditure exceeded the revenue by £108, and the Sisters of Charity are left without any maintenance fund. Dr. Youl gives the mortality of infants put out to nurse, or reared on artificial food in Victoria, as 80 per cent. The excellent directions by Dr. Stawell and Dr. Wood, of the Children's Hospital, have therefore been recently sent out to persons registered as nurses under the Infant Life Protection Act. The instructions are good, but unfortunately those most in need of them are least likely to attend to them.

### NURSING AT HYDERABAD.

Not only the hospital but the medical and nursing schools at Hyderabad are supported by the Nizam's Government. The actual buildings may appear to strangers to have little to recommend them, but they seem to satisfy the patients with whom the hospital has become very popular. The nursing department has been successfully organised by Miss Lawrie, who was trained and held the post of ward sister at the London Hospital; she has given much valuable assistance to her brother, Dr. Lawrie, since she joined him at Hyderabad and took in hand the training of native women as nurses.

### SHORT ITEMS.

The first annual report of the Pollokshaws District Nursing Association is a most encouraging one. —Two concerts, held under the patronage of Mr. and Mrs. Samuda, at Milton-under-Wychwood and at Bruern Abbey, have added substantially to the funds of the District Nursing Association.—At Southampton a successful concert has been given in aid of the local branch of the Queen's Jubilee Institute.—An excellent entertainment took place the other day at Ilkley, when some beautiful Tableaux Vivants were organised by Mrs. Herbert Bottomley, and the proceeds, £25, were handed over to St. Catherine's Home for Cancer and Incurables at Bradford.

### Elementary Anatomy and Surgery for Nurses.

By W. McAdam Eccles, M.B., M.S., F.R.C.S., Lecturer to Nurses, West London Hospital, &c.

XVI.—THE CIRCULATORY SYSTEM (*continued*).

(3) The left auricle is much like the right. It has opening into it four, sometimes only three, pulmonary veins which bring back pure blood from the lungs. (4) The left ventricle has a much thicker wall than the right and is circular in transverse section. It communicates with the left auricle by the left auriculo-ventricular opening, which is guarded by a valve having only two cusps, and therefore called the bicuspid or mitral, but which in other respects resembles the tricuspid. The largest artery of the body, known as the aorta, the commencement of the systemic circulation, leaves the ventricle at the upper and right-hand corner. (See Fig. 26.) Its orifice, like that of the pulmonary artery, presents three semilunar valves. Above these are three dilatations in the vessel, from two of which proceed the two small coronary arteries which nourish the heart itself. Externally the division between the two ventricles is marked by a groove which is apparent both in front and behind. The auricles are divided

Fig. 26.—The Left Auricle and Left Ventricle Laid Open from Behind.

*la*, Left auricle; *lv*, left ventricle; *ao*, aorta; *pa*, pulmonary artery; *mv*, mitral valve; *sv*, semilunar valves of aorta; *sv′*, the same of pulmonary artery.

from the ventricles also by depressions. The partition wall between the two auricles is called the auricular septum, and that between the two ventricles the ventricular septum. Occasionally these may, even in the adult, be not quite complete. The tip of the left ventricle forms the apex of the heart.

The relationship of the stomach beneath the diaphragm to the heart above it is one that it is important to notice and remember. The left lung with its pleura overlaps a part of the front surface of the heart. On either side of the pericardium are the lungs, the pleuræ intervening. Behind the heart will be found the æsophagus and the thoracic portion of the aorta, behind which lie the dorsal vertebræ.

This will be a convenient time to describe briefly the pulmonary and portal circulations, leaving the larger systemic circulation to be dealt with in the next lecture.

(1) The Pulmonary Circulation. (See Fig. 24 in last issue.)

The venous or impure blood is pumped by the right ventricle into the pulmonary artery. When that ventricle relaxes the blood in this artery tends to run back into the enlarging cavity of the right ventricle, but is prevented by the semilunar valves from doing so. Thus it passes on into the divisions of the pulmonary artery known as the right and left branches, proceeding to the right and left lung respectively. In the substance of the lungs the blood vessel breaks up into capillaries which are here rather larger than those of other tissues. These capillaries, being spread over the air vesicles, allow the blood to absorb oxygen from the air, and then reunite to form larger vessels, which ultimately pass back to the heart in four divisions as the pulmonary veins entering the left auricle.

(2) The Portal Circulation. (See Fig. 24.)

This is a peculiar circulation, conveying impure blood only, but which contains a large amount of nutrient material, for it is collected from practically the whole of the alimentary tract lying within the abdominal cavity. Most of the veins of the rectum, those of the sigmoid flexure, and descending colon unite to form a vein called the inferior mesenteric, which joins the splenic vein returning blood from the spleen. This latter vein unites with another large vein, the superior mesenteric, which receives from the rest of the large and the whole of the small intestine. The union of these two is called the portal vein. This, after receiving branches from the stomach, divides into two branches, one of which goes to the right and the other to the left lobe of the liver, and in the substance of that organ again undergoes division into capillaries which reuniting subsequently give rise to the hepatic veins, and these open into the inferior vena cava and so reach the right auricle of the heart. It will be seen, therefore, that the portal system has capillaries at both ends, one set in the walls of the intestines and the other in the liver substance.

### Appointments.

[It is requested that successful candidates will send a copy of their applications and testimonials, with date of election, to The Editor, The Lodge, Porchester Square, W ]

Dublin, Cork Street Fever Hospital and House of Recovery.—Miss Mary Margaret Hampson has been appointed Lady Superintendent and Matron of this institution. Miss Hampson was trained at the Nightingale School, St. Thomas's Hospital, and was a sister at St. Marylebone Infirmary, and assistant lady superintendent at Cork Street Fever Hospital. We wish her every success in her new post.

### Death in Our Ranks.

Miss Mary Eleanor Clark, who has held the post of Sister Musgrave at Bolton Infirmary since July, 1891, died of pneumonia on Sunday, April 28th. She was trained at Leeds General Infirmary, and afterwards worked at Stafford Infirmary. Miss Clark enjoyed the respect and esteem of all who knew her, and her death, in her thirty-fifth year, is deeply regretted.

### Where to Go.

Grosvenor House.—Grand concert, May 16th, at a quarter past three p.m., in aid of Woolwich Branch Church of England Soldiers' Institutes. H.R H. the Duchess of York and Prince and Princess Edward of Saxe-Weimar have promised to be present.

Grosvenor House.—May 13th, at three p.m., annual meeting of the Metropolitan and National Nursing Association.

St. George's Hall, Langham Place.—On Saturday, May 18th, a performance will be given by the Marlowe Dramatic Club in aid of the funds of the Great Northern Central Hospital.

### Dress and Uniforms.

By a Matron and Superintendent of Nurses.

### THE MIDDLESEX HOSPITAL.

The somewhat sombre exterior of the Middlesex Hospital gives but little indication of the very complete and charming nature of its internal arrangements, and perhaps less of the neat and workmanlike appearance of its nurses. The group which forms the subject of our illustration represents a sister, a nurse, and a lady probationer. The sister, who occupies a seat to the left, is attired in a uniform dress of serge of that particular violet hue known as Bishop's purple. The texture is very fine, and it is said to wear admirably. The skirt is made plain and of sufficient fulness winter grey Russell cord, of a pretty silvery shade, is worn, and in summer mauve cambric takes its place. The linen apron like that worn by the sister is ample in the skirt, and is furnished with a bib and straps that cross behind. The costume is completed by a white cambric cap, trimmed with a double row of fancy Coventry frilling, which is goffered reverse ways. Thus, one row stands upwards and the lower one rests on the hair. Two narrow strings, about a couple of inches wide, hang half way down the back. The third figure depicted in the sketch is a lady probationer. She wears a

Sister. Nurse. Lady Probationer.

NURSES AT THE MIDDLESEX HOSPITAL.

to look graceful, and is turned up round the bottom with a hem. The bodice, which is tight-fitting, fastens down the front, and is attached by a band to the skirt. A fine linen apron is worn over the dress, and this is fastened at the waist by straps that cross at the back, keeping the bib in position in front. The cap is of white cambric which fits compactly to the head. It is edged all round with a deep goffered border of Valenciennes lace, kept in position by a thread run along the reverse side and drawn sufficiently tight to form the required shape. Folded cambric strings are brought from the back of the cap and are crossed over each other on the chest. They are finished off round the bottom with a narrow piece of lace. The nurses' dress varies with the season. In black stuff dress made quite plain, and a tight-fitting bodice. The skirt is full and just clears the ground. Over this is worn a linen apron, with a deep bib and straps fastening at the back. The cap is particularly pretty, and is distinguished from all others by a delicate lavender ribbon, which encircles it at the back, and is brought behind the ears, under the chin, where it ties in a neat little bow. The cap somewhat resembles the "Marie Stuart" in shape, being pointed in front, and is made of fine muslin edged with a goffered lace border. A thread run through it at the back keeps it in the required position. The cuffs and collars consist of neat bands of linen encircling the sleeves and neck, and are identical in shape with those worn by the "sister" and "nurse."

### Hospital Secretaries at a Discount.

FEW men deserve fuller recognition for zealous, unpretentious, and most valuable public service than the secretaries of our best administered hospitals. Their remuneration is, on the whole, relatively small; the work they have to do is responsible and continuous, and they deserve the consideration of everybody. How little of this they receive, and in what estimation the secretaries of hospitals are held by some, though we hope not by many nurses, is illustrated by the following letters, which have been sent us by the secretary of one of the best administered London hospitals :—

White Cross House, 68, South Side, Clapham Common, S.W.—Dear Sir,—If at any time you should have a chronic case leaving your hospital I shall be glad to receive them into this home, and pay you commission. An answer will oblige, Yours faithfully, RITIE HARVEY.

The recipient wrote in reply that he had sent her communication to the Editor of THE HOSPITAL. In answer to this announcement the following two letters were received :

(Letter No. 1.) Sir,—I was told by Miss Prance, of 3, Knighton Park Road, whose friend was brought from your hospital to this home, that you would be glad if I would send some of my cards, as you were so often asked for a home of this kind, so I hope you will forward this letter on to the Editor of THE HOSPITAL so that he may see my reason for writing to you.—Yours truly, RITIE HARVEY.

(Letter No. 2.) Sir,—Since writing the enclosed letter I find it was to the hospital at Holloway I should have sent the cards of home. I am very sorry that I made the mistake, and hope you will accept this apology.—Yours truly, M. R. HARVEY.

It was bad enough to offer the gentleman in question a commission in the first instance, but we are inclined to think that the third letter, in which Miss Harvey implies that the hospital at Holloway (presumably the Great Northern Central Hospital) and its officials will be more amenable to the treatment she offered in the first instance to our correspondent, adds insult to injury. In China the idea that the responsible official of a great institution would be willing to accept commission in the circumstances named might have been excusable, but it is certainly remarkable that anyone familiar with the great voluntary hospitals of the British Empire should venture to make a proposal of this kind to their officers.

### Novelties for Nurses.

### KNITTED UNDERCLOTHING.

WHEN manufacturers produce such first-class commodities as Messrs. Fleming, Reid, and Co., of Greenock, it becomes unnecessary to remind those who have once become acquainted with them of their excellence. But there are a large number of people who, though well impressed with the wisdom of buying the best woollen underclothing only, are always meeting with the unpleasant experience of being possessors of inferior articles from a want of knowledge of a really good firm to supply them. Messrs. Fleming and Reid publish a list of the addresses of their branches in London and the majority of large towns. We have heard nothing but praise of articles supplied by Messrs. Fleming and Reid, and a sample card of the various qualities and styles of their knitted materials will show that what we contend as to their excellence is correct. The manufactures of this Scotch house are very varied. Besides underclothing of all kinds they make a speciality of woollen dress materials. Their catalogue is well worthy of inspection, and can be obtained from any branch house, such as 84, Oxford Street, or direct from the manufacturers at Greenock.

### Derby County Asylum.

AT the written and Oral Examinations held at the Derby County Asylum, Mickleover, on May 6th and 7th, Dr. Whitcombe, Medical Superintendent of the Birmingham Borough Asylum, examined the following attendants, all of whom obtained the certificate of proficiency in nursing of the Medico-Psychological Association: Jeremiah John Mordy, Frederick Wicks, Sarah Allen, Helen Moffat, Sarah Ann Harrison, Elizabeth Evans, and Rachel Rosa Edwards.

### A Nurses' Pension Fund for Sweden.

THE great interest which Her Majesty the Queen of Sweden has always exhibited in the progress of nursing, and in the advancement of the personal welfare of nurses, is far too widely realised to need comment. The scheme for establishing a national pension fund for nurses in Sweden cannot fail to secure the powerful support of Her Majesty as it has already attracted her interest.

In the course of the summer it is the intention of those who have the subject at heart to place before the public an outline of the Royal National Pension Fund, which will enable the Press to put forward the substantial benefit to the country as well as to individual nurses of organising a similar fund in Stockholm.

Many eminent physicians and other influential persons have promised their support, and the inauguration of a "Donation Fund" is spoken of with confidence. Widely spread interest has been already exhibited, not only by the nurses of Sweden but also by the public in general, and the Press will find a large audience eagerly receptive of its promised details.

Under such encouraging auspices, and with the guarantee of Royal patronage which is doubtless forthcoming, the pension fund for the nurses of Sweden ought to be a well-established institution in the course of the autumn. Most cordial and courteous acknowledgment has been already made respecting the aid which England has given to Sweden by placing full particulars of the working of the Royal National Pension Fund for Nurses at the disposal of the ladies at Stockholm. The able and comprehensive scheme, easily elucidated from Mr. Dick's exhaustive details, appears to have gained such admiration and appreciation as its merits deserve. English policy-holders rejoice to find the benefits enjoyed by themselves are likely to be offered on similar lines to their sister nurses in Sweden.

The "sincere flattery" of imitation will become so widespread, that in a few years it will doubtless be impossible to find any country without some such Royal National Pension Fund as the one of which H.R.H. the Princess of Wales is the active President, liberally supported by the great capitalists of England.

### Everybody's Opinion.

[Correspondence on all subjects is invited, but we cannot in any way be responsible for the opinions expressed by our correspondents. No communications can be entertained if the name and address of the correspondent is not given, or unless one side of the paper only be written on.]

### HOLIDAYS.

NURSE MARY writes: The Cheshire district nurse whose letter in THE HOSPITAL of June 23rd, 1894, brought many nurses to enjoy the rest and pleasant home there offered for small charge, will be glad to again receive nurses subject to same conditions. Terms, and all particulars, may be had by writing to Nurse Mary, 12, Teddington Park Road, Teddington, Middlesex. A stamped addressed envelope should accompany all applications. Two friends will be received on reduced terms if willing to share rooms.

### THE EMPLOYER'S POINT OF VIEW.

"A NURSE" writes from Ireland: I have read in THE HOSPITAL a good deal of discussion as to the work of private nurses. The point as to where a nurse should take her meals was discussed in a recent issue; one nurse says "many excellent private nurses belong to the servant class." Now, I believe "an excellent nurse" must have better education than is common in the class referred to; but if all private nurses belonged to it, *à fortiori* they ought not to take their meals with the servants, unless the patient and family wish the patient's symptoms and all his little weaknesses talked over in the kitchen. A lady sitting down with the servants would not be likely to converse freely with them on such subjects. Although a nurse myself, I have had occasion to look at this question also from the employer's point of view.

### HORNSEY ISOLATION HOSPITAL.

The Chairman of the Hospital Committee of the Hornsey Urban District Council, writes: My attention has been called to a paragraph in The Hospital of last week, headed "Night Nursing Sanctioned," in which it is stated that the Hornsey Hospital Committee consider that they ought to employ a night nurse, and that the recommendation has been carried by the Council. The fact is, that either a nurse on the permanent staff or a nurse temporarily engaged has in all cases been in charge at night time of any ward in which there has been a patient requiring attention. The only change made is that, in consequence of the erection of new buildings an additional nurse has been engaged on the permanent staff expressly for night duty. Your insertion of this letter in this week's issue of The Hospital will oblige.

*** From the above statement, that a nurse "has in all cases been in charge at night time of any ward in which there has been a patient requiring attention," we gather, as we have already stated, that there has hitherto been no regular night nursing. We congratulate the hospital committee in the reform they have introduced.—Ed. *T. H.*

### PRIVATE NURSING.

"A Subscriber" writes: "L. M. R." seems to be still of the opinion that we ought to be stuck in the class of "Domestic Maids." I do not think an educated nurse, whose ideas and manners soar above the generality of servants, would care to come continually in contact with domestics whose conversation runs chiefly on "young men." If a nurse cannot have her meals with the family, she should have them alone, where she can make use of a few spare moments in reading or thinking. I do not think it degrades a nurse to do a little house-work; and in monthly nursing there must be a certain amount of it where only one "maid" is kept.

"M. G." writes: I think the experience of most private nurses will teach them that there can be no hard and fast rule concerning their duties, but that they must adapt themselves to the circumstances of each household to which they are called. If a private nurse finds herself in a family of limited means, where only one servant is kept, she should cheerfully do all she can in the sick-room to lighten the duties of the maid, and thereby also assure herself that everything is thoroughly done. If her next case is with wealthy people, and several servants, she will do well to confine her attention solely to the personal care of the patient, for should she insist on excluding the servants from their usual duties, she will probably soon find that she has given herself a great deal of trouble, and that her well-meant efforts are totally unappreciated by both mistress and maids. Of course this does not apply to infectious cases; then it goes without saying that no servant must be admitted, and that the nurse must do everything necessary in the sick room. In no good hospital is a nurse asked to take her meals with the ward maids, or to do ward maids' work. Why, then, should it be deemed needful to do such things in the supposed superior refinement of a private house? If it is not desirable that the nurse should have her meals with the family, they should be nicely served for her in a room alone. The public usually show sufficient discernment in this, and if not, it should be instilled into them. If the modern nurse is superior to one of fifty years ago, she should receive superior consideration.

### INDIAN NURSING SISTERS.

"Our Indian Correspondent" writes: The Chitral Expedition has plunged the North of India into terrific excitement; the majority of British regiments stationed in the Punjab are already at the front. All leave has been stopped for hospital attendants and for the medical staff. Numbers of doctors have been despatched to the front, and five extra nursing sisters (in addition to four already there) have been told to hold themselves in readiness for the hospital at the Base. It is the biggest expedition out here since the Afghan war. Numbers of the sisters have volunteered for the front, but the chief has been obliged to harden his heart against all applications. So far no sisters are actually to go with the field force, the difficulties of transport are so great; even officers are not allowed tents and thirty pounds of baggage only are permitted for each officer. Colonel Kelly's party are fighting in the snow, and General Kinloch's brigade pushing on to them.

### Notes and Queries.

### Queries.

(132) *Appointment.*—Can you tell me who has been appointed Matron of the Cork Street Fever Hospital, Dublin? I wrote for particulars of the vacant post, and sent a stamped envelope for reply, but have not received one.—*A. H.*

(133) *Male Nurse.*—Where can a man of 53, healthy, strong, sober, quiet, and well educated get trained as a nurse-attendant? If there is no place where he could get this instruction, do you think the man could get employed as a nurse attendant at any institution or in a private family? He has had no experience.—*Nurse Florence.*

(134) *Mental Nursing.*—Where can I get instruction in mental nursing?—*K. K.*

(135) *Nursing.*—Having received three years' training, I want to go into a hospital abroad, and shall be glad of advice.—*Nurse T.*

(136) *Sailors.*—In what hospitals are sailors nursed?—*Nurse H.*

(137) *Work.*—I am not strong enough for hospital training and want to take up work in the neighbourhood. Can you advise me as to nursing books?—*E. B. C.*

(138) *India.*—I should be deeply grateful for information regarding nursing in India.—*Doreen.*

(139) *Training.*—I am strong, healthy, and educated, and want advice as to being trained for a nurse.—*T. G.*

(140) *Probationer.*—Please tell me how to get trained, and if I could begin soon?—*R.*

(141) *Mental Nursing.*—Kindly tell me the best way to obtain training in mental nursing.—*A. M. A.*

### Answers.

(132) *Appointment (A. H.).*—Your question is answered under "Appointments" in this week's issue of The Hospital.

(133) *Male Nurse (Nurse Florence).*—There is no such training in general nursing provided for men in England. We imagine that age would be considered a drawback for asylum work. If a doctor who knows the man chooses to put him in charge of a patient he would, of course, take all responsibility. It is obvious that without training or experience a man cannot be called a "nurse attendant." As a nurse yourself you must realise that a man needs training no less than a woman. We advise some other branch of work for one whose character appears satisfactory, though he is ignorant of the elements of the calling you suggest he should follow.

(134) *Mental Nursing (K.K.).*—You had better apply to the medical superintendent of Berrywood Asylum, Northampton, for terms and regulations. Ladies are also taken for training at Bethel, Norwich. You can obtain particulars from the lady superintendent, who is a trained nurse.

(135) *Nursing (Nurse T.).*—You should not think of going abroad except to a definite appointment. So many colonies train their own nurses now, and therefore Englishwomen are not very cordially received. You will see advertisements of vacant posts from time to time in our columns.

(136) *Sailors (Nurse H.).*—In all hospitals on the coast you will find sailors. At the Seamen's Hospital, Greenwich; at Poplar Accident Hospital; at the London Hospital, Whitechapel, and others

(137) *Work (E. B. C.).*—Miss Lücke's "Lectures to Nurses" is excellent, also Lewis's "Theory and Practice of Nursing," and Burdett's "Help in Sickness." You would find much valuable information in the set of articles on "District Nursing," which appeared in The Hospital last year, commencing in January. A few months spent in a provincial hospital would teach you a great many practical points.

(138) *India (Doreen).*—The Indian Nursing Service, for which a form of application can be obtained at the India Office, St. James's Park, S.W. Particulars of "Up-Country Nursing Association for Europeans in India" can be obtained through the Hon. Mrs. Neville Lyttelton, 21, Carlton House Terrace, hon. treasurer, or Major-General J. Bonus, R.E., The Cedars, Strawberry Hill, hon. sec.

(139) *Training (T. G.).*—Get "How to Become a Nurse," published by the Scientific Press, 428, Strand, London. Why do not you call and see the matrons of the large Liverpool and Manchester Hospitals, and get forms of application.

(140) *Probationer (R.)*—See other answers this week. There are more vacancies at infirmaries than hospitals just now, and the training is good in some of them. You might write to the matrons of St. Marylebone Infirmary, Notting Hill, or St. Pancras Infirmary, Highgate, or Lewisham Infirmary. They are adding to the staff of the London Hospital, Whitechapel, so you might have a chance there if you applied at once.

(141) *Mental Nursing (A.M.A.).*—See our reply to Query No. 134 above.

## Figures and Tables

**Fig. 26 f1:**
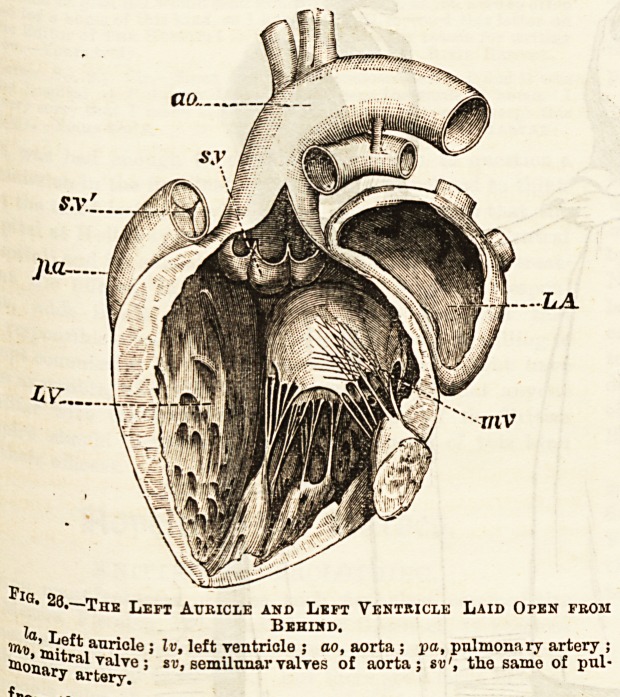


**Figure f2:**